# Assessment of Breast Milk Iodine Concentrations in Lactating Women in Western Australia

**DOI:** 10.3390/nu8110699

**Published:** 2016-11-04

**Authors:** Anita Jorgensen, Peter O’Leary, Ian James, Sheila Skeaff, Jillian Sherriff

**Affiliations:** 1 School of Public Health, Curtin University, Perth 6102, Australia; j.sherriff@curtin.edu.au; 2 Faculty of Health Sciences, Curtin University, Perth 6102, Australia; peter.oleary@curtin.edu.au; 3 Institute for Immunology & Infectious Diseases, Murdoch University, Murdoch 6150, Australia; i.james@murdoch.edu.au; 4 Department of Human Nutrition, University of Otago, Dunedin 9054, New Zealand; sheila.skeaff@otago.ac.nz

**Keywords:** iodine, breast milk, supplementation, iodine status

## Abstract

Breast-fed infants may depend solely on an adequate supply of iodine in breast milk for the synthesis of thyroid hormones which are essential for optimal growth and cognitive development. This is the first study to measure breast milk iodine concentration (BMIC) among lactating women in Western Australian (*n* = 55). Breast milk samples were collected between 2014 and 2015 at a mean (±SD) of 38.5 (±5.5) days post-partum. The samples were analysed to determine median BMIC and the percentage of samples with a BMIC < 100 µg/L, a level considered adequate for breast-fed infants. The influence of (a) iodine-containing supplements and iodised salt use and (b) consumption of key iodine-containing foods on BMIC was also examined. The median (p25, p75) BMIC was 167 (99, 248) µg/L and 26% of samples had a BMIC < 100 µg/L. Overall, BMIC tended to be higher with iodine-containing supplement usage (ratio 1.33, 95% confidence interval (CI) (1.04, 1.70), *p* = 0.030), cow’s milk consumption (ratio 1.66, 95% CI (1.23, 2.23), *p* = 0.002) and lower for Caucasians (ratio 0.61, 95% CI (0.45, 0.83), *p* = 0.002), and those with secondary school only education (ratio 0.66, 95% CI (0.46, 0.96), *p* = 0.030). For most women, BMIC was adequate to meet the iodine requirements of their breast-fed infants. However, some women may require the use of iodine-containing supplements or iodised salt to increase BMIC to adequate levels for optimal infant nutrition.

## 1. Introduction

Iodine, an essential nutrient, is required by humans for the synthesis of thyroid hormones which are vital for normal growth and development [1,2]. A regular and adequate supply of iodine is particularly important during the critical period for brain and central nervous system development, namely, from the second trimester of pregnancy to 3 years of age [1]. Iodine deficiency during this time results in a spectrum of adverse effects known as iodine deficiency disorders, with the most severe outcomes, irreversible mental impairment and cretinism, resulting from severe iodine deficiency during pregnancy. In infants, iodine deficiency leading to inadequate thyroid activity results in delayed growth and physical development, and impaired cognitive function [1,2].

During intrauterine life, iodine is transferred from the mother to the fetus [3]. This results in a pool of iodine stored in the fetal thyroid gland, with the size of the pool strongly reflecting maternal dietary iodine intake. However, even under conditions of maternal iodine sufficiency, this fetal iodine pool is small and turns over rapidly after birth to partly support the iodine demand of newborns [4]. Infants, however, rely solely on dietary sources to meet their iodine needs. Breast-fed infants are particularly vulnerable to iodine deficiency as they may be completely dependent on the iodine concentration of breast milk for their intake of iodine [5]. Consequently, maternal iodine requirements are increased during breastfeeding to provide sufficient amounts for the mother and to also meet the iodine demands of the developing infant, via breast milk. Given that 40%–45% of the iodine ingested by the mother appears in breast milk [6], a maternal iodine intake during breastfeeding of 190 µg/day (Australian Estimated Average Requirement (EAR)) would provide just under the Australian Adequate Intake (AI) of 90 µg/day for infants aged 0–6 months [7]. This is achieved by a physiological response during breastfeeding whereby iodine is strongly concentrated by the lactating mammary gland due to the increased expression of the sodium iodide symporter, the main iodine transporter in lactating breast cells [8]. This results in human milk having an iodine concentration 20–50 times higher than that of plasma [4].

Breast milk iodine concentration (BMIC) is influenced by, and may be an indicator of, maternal iodine status during breastfeeding [4,5,8,9,10,11]. BMIC is also influenced by other factors including recent maternal iodine intake [12] and duration of lactation [5]. While no reference ranges for the adequate iodine concentration of breast milk have been specified, values above 75 µg/L have been suggested to indicate sufficient maternal iodine intake [4]. An iodine balance study of full-term infants found that a positive iodine balance is only achieved when iodine intake is 15 µg/kg per day, which equates to a BMIC of 100–200 µg/L [8].

A wide range of median or mean BMIC values has been reported in several reviews conducted in areas of varying iodine sufficiency [4,8,10]. BMIC typically ranges from <50 µg/L in iodine-deficient areas [5] to 100–150 µg/L in areas of iodine sufficiency [4] and as high as 150–180 µg/L in areas of good iodine supply [8,10]. A BMIC < 100 µg/L has been identified in studies from France, Germany, Belgium, Sweden, Spain, Italy, Denmark, Thailand and Zaire while studies from Iran, China, USA and some parts of Europe have identified above this level [4]. A recent study in Nepal identified a median BMIC of 250 µg/L, and the estimated iodine intake of the infants involved (0–6 months) was 200 µg/day [13]. WHO’s recommended maximum iodine intake for infants <2 years old is 180 µg/day [14], therefore some infants in this area may be consuming excessive iodine intakes through breast milk [13]. This can result in subclinical hypothyroidism and permanently affect their neurodevelopment [15].

In recent decades, Australia has been regarded as a country with mild iodine deficiency. Two initiatives introduced in response to the re-emergence of this public health issue are the mandatory fortification of all bread (except organic) with iodised salt in 2009 [16] and the 2010 National Health and Medical Research Council recommendation that all pregnant and breastfeeding women take a daily supplement containing 150 µg of iodine [17]. Despite this recommendation, only two studies have examined the iodine content of breast milk in Australia to assess either iodine provision to breastfed infants or maternal iodine status. The first was a small (*n* = 50) cross-sectional study of breastfeeding women in Sydney, conducted more than a decade ago and prior to mandatory iodine fortification. This study identified a median BMIC of 84 µg/L [18], indicating inadequate maternal iodine intake based on the adequate cut-off of 100 µg/L. The second larger and more recent study compared the BMIC of lactating women in South Australia pre- (*n* = 291) and post- (*n* = 653) mandatory fortification. The median BMIC of samples from both periods were indicative of adequate breast milk iodine levels, however, BMIC was significantly higher in the post-fortification samples compared with the pre-fortification samples (187 vs. 103 µg/L; *p* < 0.05) [19].

To date, there is no information regarding BMIC for lactating mothers in Western Australia (WA), nor for iodine status of WA breastfeeding women. WA has long been considered an iodine-sufficient area of Australia, based on measures of iodine status in studies involving school-children and adults [20,21]. However, this outcome may not reflect the iodine status in breastfeeding women, who have substantially greater requirements for iodine [1]. In the present study we examined BMIC in breastfeeding women in a local cohort to determine adequacy of iodine provision to breastfed infants. We also investigated the influence of iodine-containing supplements and iodised salt use, as well as the consumption of key iodine-containing foods, on this biomarker of iodine status.

## 2. Materials and Methods

### 2.1. Subjects and Design

Participants were recruited in 2013–2014 as part of the Perth Iodine and Pregnancy Study II (PIPS II) via advertising (flyer in private women’s ultrasound practices *n* = 15 and newspaper, radio and websites *n* = 7), in-person by study coordinator (public maternity hospital antenatal clinics *n* = 21 and pathology centre *n* = 3) and word of mouth (*n* = 8). At the time of recruitment, women were aged 18 years and over and were in the first or second trimester of pregnancy (gestation range 5–22 weeks). Other inclusion criteria were no history of thyroid disease, not currently taking thyroid medication, having a singleton birth and not currently breastfeeding but with the intention to breastfeed their baby. Women were excluded from the study if English was not the main language spoken at home. The study was approved by the Curtin University (Approval No. HR 47/2013; 15 April 2013) and Women and Newborn Health Service Human Research Ethics Committees (Approval No. 2014075EW; 4 August 2014) and informed written consent was obtained from each participant.

Breast milk samples were collected between February 2014 and August 2015. Participants were mailed vials for sampling together with instructions to provide (duplicate) 5 mL nonfasted breast milk samples at home at the start of a single morning feed (preferably between 0900 and 1200 h) when their baby was aged 4–6 weeks. Women were asked to record baby’s age and time of day of sampling. Participants were also asked to provide information on current medication use, daily use of dietary supplements, daily intake (yes/no) of any amount of six key iodine-containing foods (cow’s milk, cheese, ice cream, yoghurt, bread/bread products, eggs), use of iodised salt (yes/no) and whether or not they smoke cigarettes. Sociodemographic characteristics of the women, namely parity, age, postcode, ethnicity, household income and education, had been collected previously.

### 2.2. Laboratory Procedures

Breast milk samples were stored at −20 °C from time of sampling until collection and then at −80 °C until analysis. After thawing, milk samples were homogenized before analysis by inductively coupled plasma mass spectrometry (ICPMS) in an accredited commercial laboratory (PathWest Laboratory Medicine WA, Nedlands, Australia). ICPMS is considered the gold standard to determine iodine concentration in complex sample matrices such as breast milk [9,22]. An optimised ICPMS method for breast milk has been published recently [22] and was adapted for this study. In brief, sonicated breast milk samples were diluted in mild alkali solution, ionized with inductively coupled plasma and the ions separated and quantified in a Perkin Elmer NexION 300 ICP-MS mass spectrometer (PerkinElmer Inc., Waltham, MA, USA).

### 2.3. Statistical Analysis

Distributions of BMIC were skewed and descriptive statistics reported as medians and 25th, 75th percentile. The proportion of women with BMIC < 100 µg/L, the suggested cut-off for providing an adequate iodine supply to breast-fed infants, was also determined. Statistical analyses of BMIC were carried out on the log base 10 scale to better approximate normality. Multiple regression analyses were used to assess associations of BMIC with: (a) use of iodine supplements and iodised salt; (b) daily consumption of six key iodine-containing foods (yes/no); and (c) all studied factors simultaneously. Associations of cohort characteristics with use of iodised salt or iodine supplements were assessed via logistic regressions. Data were analysed using IBM SPSS version 20 (IBM Corporation, Tokyo, Japan) and TIBCO Spotfire S+ version 8.2 (TIBCO Software Inc., Boston, MA, USA). A 5% level of significance was chosen.

## 3. Results

**Figure 1:**
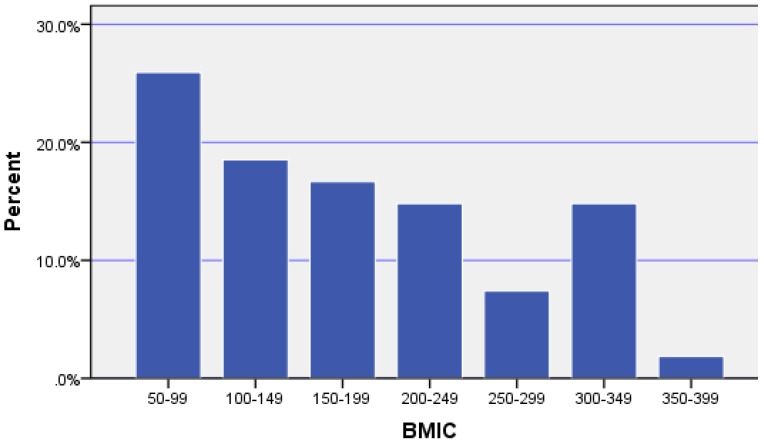
Percentage distribution of breast milk iodine concentration (BMIC) (µg/L).

Sociodemographic characteristics of the 55 study participants are shown in Table 1. The mean age (±standard deviation (SD)) of the study women was 31.4 (±4.7) years. This is consistent with the average age of women who gave birth in WA in 2013 of 29.8 years [23]. The majority of women were pregnant for the first time (52.7%), were tertiary educated (72.3%), had a total household income of >$AUS100K (67.3%) and were Caucasian (80.0%). Compared with available Western Australian data from the Australian Bureau of Statistics Census 2011, our cohort included an over representation of women with a higher reported education level and higher household incomes [24]. All women provided breast milk samples, however one woman was excluded as she reported being a smoker, thus leaving 54 women for breast milk analysis. Breast milk samples were collected at 28–56 days postpartum with a mean (±SD) of 38.5 (±5.5) days. All samples were provided in the morning between 0600 h and 1200 h. The median (p25, p75) BMIC was 167 (99, 248) µg/L indicating adequate maternal iodine intake for the group. However, 26% of women had BMIC less than the suggested cut-off level for adequacy of 100 µg/L (see Figure 1).

Thirty-one women (57.4%) reported the daily use of an iodine-containing supplement, some of which contained less than the amount recommended (i.e., 150 µg). Use of iodised salt and iodine-containing supplements were independently associated with increases of similar magnitudes in BMIC (ratio 1.37, 95% CI (1.05, 1.80), *p* = 0.025 and ratio 1.37, 95% CI (1.04, 1.79), *p* = 0.029, respectively—see Table 2). There was no significant difference (*p* = 0.96) between the median BMIC values for the use of either iodised salt or iodine-containing supplements without the other. Among all cohort characteristics jointly considered only low household income (<$AUS50K) remained significantly (negatively) associated with iodine supplement usage (1/8 vs. 30/46, *p* = 0.010 Fisher test). Exactly half of the women reported using iodised salt, with usage higher among non-Caucasians (10/11 vs. 17/43, *p* = 0.005).

For the six key iodine-containing foods, the majority of women reported daily consumption of bread/bread products (79.6%) and cow’s milk (77.8%) and with just less than half of women (46.3%) reporting daily intake of cheese. Furthermore, about a third of women (37.0%) reported daily consumption of yoghurt, around a quarter (27.8%) ate eggs daily and 5.6% of women said they ate ice cream each day. However, only daily cow’s milk intake was significantly associated with higher BMIC values after adjusting for the other foods or on its own (ratio 1.44, 95% CI (1.01, 2.06), *p* = 0.49 and ratio 1.44, 95% CI (1.03, 2.01), *p* = 0.040, respectively). None of the other foods were significant, jointly or marginally, in influencing BMIC values. Furthermore, cow’s milk remained the only food positively associated with BMIC after adjustment for iodine-containing supplements and iodised salt use (ratio 1.50, 95% CI (1.10, 2.04), *p* = 0.013). Overall in the joint model, BMIC tended to be higher with iodine-containing supplement usage and cow’s milk consumption and lower for Caucasians and those with secondary school only education (see Table 3).

## 4. Discussion

This is the first study to report BMIC values for breastfeeding women in Western Australia. The median BMIC value of women would provide an adequate iodine supply for breastfed infants. However, BMIC levels were below the suggested adequate cut-off (100 µg/L) for 26% of women, indicating some infants may be at risk for iodine deficiency, especially if exclusively breast-fed as is recommended. These findings are consistent with results for the post-fortification cohort of the recent South Australian study. However, compared to our study, the proportion of women with BMIC below the adequate cut-off level was considerably lower in the study by Huynh et al. (26% vs. 13%) [19]. The one participant who reported being a smoker in the present study was excluded from breast milk analysis as the chemical thiocyanate found in cigarettes competitively inhibits the sodium iodide transporter in the lactating breast and impairs iodine transport into breast milk [25], thereby distorting BMIC values.

Despite the NHMRC recommendation for all breastfeeding women to use a daily 150 µg iodine supplement, only about half of the women (54%) in the present study reported behaviour consistent with this (an additional two women reported use of a daily iodine supplement containing less than the recommended iodine amount). In a recent study of breastfeeding women conducted in regional New South Wales (*n* = 60), iodine-containing supplements were being taken by 45% of women, although frequency of use and iodine content were not documented [26]. These results suggest a low level of awareness and/or compliance amongst Australian breastfeeding women regarding the national iodine supplement recommendations. In contrast, 90% of South Australian women in the post-fortification cohort reported use of supplements containing any iodine [19], although again details of frequency of use and iodine content were not documented. Furthermore, given the low use of iodine-containing supplements in low income cases compared to higher income participants (12.5% vs. 65.2%, respectively) in the present study, perhaps the availability of government subsidized iodine supplements is warranted in Australia, as is the case in New Zealand. Interestingly, in the Perth Infant Feeding Study Mark II conducted in 2002–2003 prior to the supplement recommendation, no breastfeeding women reported taking iodine supplements [27].

In addition, 50% of women in the present study reported using iodised salt. This is similar to the 45% of lactating women using iodised salt in the regional New South Wales study by Charlton et al. [26]. In the present study, use of iodised salt was significantly higher among non-Caucasians (*p* = 0.013), possibly explaining why BMIC tended to be higher in non-Caucasian mothers compared with Caucasian mothers (*p* = 0.002). This later finding is consistent with the results of the South Australian study by Huynh et al. [19].

As shown in Table 2, use of both iodine-containing supplements and iodised salt together resulted in the highest median BMIC value (272 µg/L). The use of either iodine-containing supplements or iodised salt had similar positive effects on median BMIC values, suggesting both methods are equally effective in improving the iodine content of breast milk. Our results are consistent with other recent studies that have examined the effect of supplementation and/or iodised salt use on breast milk iodine content [28,29]. The lowest median BMIC was recorded for those women using neither iodine-containing supplements nor iodised salt. This median BMIC value of 98 µg/L is borderline for inadequate BMIC using the cut-off of 100 µg/L. This suggests that for women in our study, food sources alone may not provide the amounts of iodine required during breastfeeding to meet maternal and infant needs. Furthermore, given breast milk samples in the present study were provided in the early post-partum period and BMIC of iodine-deficient lactating women has been shown to decrease in the first 6 months postpartum [5], the use of some form of iodine supplementation by these women is important.

Of the six key iodine-containing foods examined in the study, only daily cow’s milk consumption was significantly associated with higher BMIC values, independent of other foods and supplement and iodised salt use. Some cow’s milk was consumed daily by more than three-quarters of women in the study. Despite quantity not being examined in the present study, this suggests the importance of cow’s milk consumption in terms of iodine intake for breastfeeding women. Interestingly, milk and dairy foods were the highest contributors to iodine intake in the study by Charlton et al. which used a self-administered validated iodine-specific food frequency questionnaire to assess dietary iodine intake of Australian breastfeeding women [26]. Conversely, daily consumption of bread/bread products was not associated with higher BMIC values, despite the fact that a very high proportion of women reported consumption of these foods daily and their known fortification with iodine. This finding therefore questions the impact of the bread fortification initiative for lactating women in relation to BMIC.

There are some limitations to the interpretation of our study findings. Firstly, the impact of time of supplement intake, iodised salt use and consumption of key iodine-containing foods relative to breast milk sampling were not examined. Leung et al. [12] reported a rise in BMIC following acute oral ingestion of 600 µg potassium iodide, with peak levels at 6 h post-ingestion, and concluded that recent maternal iodine intake would influence the interpretation of BMIC values. Furthermore, as BMIC values fluctuate throughout the day, single breast milk samples provide an imprecise measurement of daily iodine output or maternal iodine sufficiency [30]. In addition, actual compliance with reported supplement use, use of iodised salt or intake of foods examined in the 24-h prior to breast milk sampling could not be confirmed with participants. Finally, while the study included a cross-section of breastfeeding women from both public and private health care systems, the sample size is relatively small and all women who participated (bar one) lived in the Perth metropolitan area, so generalisability of results to the wider breastfeeding population is made with qualifications.

## 5. Conclusions

Despite these limitations, for the majority of women in the present study, BMIC was adequate to meet the iodine requirement of their breast-fed infants. However, the study also indicates that some breast-fed infants may be at risk of iodine deficiency, which could potentially be reduced by the maternal use of iodine-containing supplements and/or iodised salt. Further studies of women representing the social and regional diversity of the population will be needed to confirm our findings.

## Figures and Tables

**Table 1:** Sociodemographic characteristics of study participants (*n* = 55).

	*n*	%
First pregnancy		
Yes	29	52.7
No	26	47.3
Highest education ^1^		
Secondary school	7	12.7
Trade or technical	3	5.5
Diploma	5	9.1
Professional	2	3.6
Bachelor degree	22	40.0
Postgraduate university	16	29.1
Total household income		
<$AUS50K	8	14.5
$AUS50–100K	8	14.5
>$AUS100K	37	67.3
Don’t wish to answer	2	3.6
Ethnicity		
Caucasian	44	80.0
Non-Caucasian	11	20.0

^1^ Tertiary educated includes Professional, Bachelor degree and Postgraduate university.

**Table 2:** Effect of iodine supplement and iodised salt use on BMIC.

	*n*	Median BMIC (µg/L)
Yes supplement + Yes salt	15	272 **
Yes supplement + No salt	16	151 *
No supplement + Yes salt	12	156 *
No supplement + No salt	11	98 **

Overall *p* = 0.028; * There was no difference between the ‘Yes supplement + No salt’ and ‘No Supplement + Yes salt’ groups (*p* = 0.960); ** There was a significant difference between the ‘Yes supplement + Yes salt’ and ‘No supplement + No salt’ groups (*p* = 0.003).

**Table 3:** Significant joint explanatory variables for BMIC *.

Variable	Ratio ** (95% CI)	*p*-Value
Caucasian ethnicity	0.61 (0.45, 0.83)	0.002
School only education	0.66 (0.46, 0.96)	0.030
Iodine supplement use	1.33 (1.04, 1.70)	0.030
Cow’s milk consumption	1.66 (1.23, 2.23)	0.002

* Analyses carried out on the log BMIC scale with non-significant terms (sociodemographic and dietary factors) removed by backwards elimination; ** Exponentiated coefficient from the joint model for log (BMIC) predicts the ratio of BMIC for the listed category relative to those not in the category, given fixed values of the other variables.
